# Comparing Once- versus Twice-Weekly Yoga Classes for Chronic Low Back Pain in Predominantly Low Income Minorities: A Randomized Dosing Trial

**DOI:** 10.1155/2013/658030

**Published:** 2013-06-26

**Authors:** Robert B. Saper, Ama R. Boah, Julia Keosaian, Christian Cerrada, Janice Weinberg, Karen J. Sherman

**Affiliations:** ^1^Department of Family Medicine, Boston University School of Medicine and Boston Medical Center, 1 Boston Medical Center Place, Dowling 5 South, Boston, MA 02118, USA; ^2^Department of Biostatistics, Boston University School of Public Health, Boston, MA 02118, USA; ^3^Group Health Research Institute, Group Health Cooperative, Seattle, WA 98112, USA; ^4^Department of Epidemiology, University of Washington, Seattle, WA 98195, USA

## Abstract

*Background*. Previous studies have demonstrated that once-weekly yoga classes are effective for chronic low back pain (cLBP) in white adults with high socioeconomic status. The comparative effectiveness of twice-weekly classes and generalizability to racially diverse low income populations are unknown. *Methods*. We conducted a 12-week randomized, parallel-group, dosing trial for 95 adults recruited from an urban safety-net hospital and five community health centers comparing once-weekly (*n* = 49) versus twice-weekly (*n* = 46) standardized yoga classes supplemented by home practice. Primary outcomes were change from baseline to 12 weeks in pain (11-point scale) and back-related function (23-point modified Roland-Morris Disability Questionnaire). *Results*. 82% of participants were nonwhite; 77% had annual household incomes <$40,000. The sample's baseline mean pain intensity [6.9 (SD 1.6)] and function [13.7 (SD 5.0)] reflected moderate to severe back pain and impairment. Pain and back-related function improved within both groups (*P* < 0.001). However, there were no differences between once-weekly and twice-weekly groups for pain reduction [−2.1 (95% CI −2.9, −1.3) versus −2.4 (95% CI −3.1, −1.8), *P* = 0.62] or back-related function [−5.1 (95% CI −7.0, −3.2) versus −4.9 (95% CI −6.5, −3.3), *P* = 0.83]. *Conclusions*. Twelve weeks of once-weekly or twice-weekly yoga classes were similarly effective for predominantly low income minority adults with moderate to severe chronic low back pain. This trial is registered with ClinicalTrials.gov NCT01761617.

## 1. Introduction

Chronic low back pain (cLBP) is a significant source of morbidity, disability, and health care cost. Four large (*n* = 90–313) [1–4] and five smaller randomized controlled trials (RCTs) (*n* = 20–80) [5–9] have demonstrated yoga is an effective treatment for reducing pain and improving function in adults with cLBP. Meta-analyses [10–12] and practice guidelines from the American Pain Society and American College of Physicians [13] support yoga as an evidence-based treatment for cLBP with at least moderate benefit.

Complementary alternative medicine (CAM) modalities with evidence of benefit for specific conditions should be studied to determine optimal dose and generalizability to diverse populations [3, 14, 15]. However, few nonpharmacologic CAM trials [16–18] and even fewer yoga studies [19, 20] have rigorously evaluated dose. Several variables can impact yoga dose, such as frequency, duration, and content of classes; duration of intervention; and home practice. For cLBP, the majority of yoga studies have used a 12-week regimen of one 75-minute class per week supplemented by home practice [20]. For this randomized dosing trial of yoga for cLBP, we chose to vary weekly class frequency, as this was feasible to implement, could be reliably measured, and appeared to be an efficient method for increasing total time spent doing yoga. Thus, our primary study objective was to determine if 12 weeks of twice-weekly 75-minute classes provided superior benefit in low back pain intensity and back-related function compared to 12 weeks of once-weekly 75-minute classes.

Furthermore, most yoga for cLBP studies [1–5, 7, 21] enrolled predominantly white well-educated adults with high socioeconomic status. However, our 2009 pilot yoga for cLBP RCT suggested recruitment of lower income minorities was feasible [6]. A secondary objective of the current study, therefore, was to determine if yoga's improvement for cLBP was generalizable to a racially diverse, lower income population. Lastly, this study was planned to inform the dose and design of a future larger and longer comparative effectiveness trial of yoga, physical therapy, and education for cLBP.

## 2. Methods

### 2.1. Design Overview

We conducted a 12-week two-group parallel randomized dosing trial for persons with nonspecific chronic low back pain. The Boston University Medical Campus Institutional Review Board, Boston HealthNet Research Committee, and individual community health center research committees approved the study.

### 2.2. Setting and Participants

From May to December 2011, recruitment and classes took place at Boston Medical Center, an academic safety-net hospital, and five affiliated federally qualified community health centers. These centers are part of a 15-site integrated health care delivery system (Boston HealthNet) located throughout diverse Boston neighborhoods.

To recruit participants, we mailed study invitation letters to adult patients who made visits to any of the sites and had associated ICD-9 diagnosis codes 724.2 (lumbago) and 724.5 (backache unspecified) in the previous two years. The research team also made presentations to clinic staff and placed flyers throughout the health centers. Each site had a physician “study champion” to assist with recruitment and liaise with study staff.

Inclusion criteria were being 18–64 years old; current nonspecific low back pain [22] persisting ≥12 weeks; having average low back pain intensity ≥4 for the previous week on an 11-point numerical rating scale where 0 = no pain and 10 = worst possible pain; and sufficient English fluency to understand yoga class instructions and complete questionnaires. We excluded individuals with known specific back pain pathologies (e.g., spinal canal stenosis, spondylolisthesis, ankylosing spondylitis, severe scoliosis, malignancy, and fracture); sciatica pain equal to or greater than low back pain; back surgery in the previous three years; severe or progressive neurological deficits; new back pain treatments started within the previous month or anticipated to begin during the study; pregnancy; yoga practice in the previous six months; active or planned worker's compensation, disability, or personal injury claims; or perceived religious conflict.

After obtaining verbal consent, we conducted eligibility screening using computer-assisted telephone interviews via an electronic data capture system (StudyTrax, Macon, GA). We obtained permission to review medical records and speak with patients' physicians if their eligibility was uncertain. Potentially eligible individuals then attended an in-person visit at their health center to learn more about the study, and if desired, provide written informed consent.

### 2.3. Randomization and Interventions

In the week prior to the onset of yoga classes, consented participants returned to their health center sites to complete baseline surveys (see Section 2.4 below). Participants were then randomized and informed of their group assignment. The 1 : 1 randomization schedule was created in StudyTrax using a permuted block design with randomly determined block sizes (4, 8, 12). The unit of randomization was the participant, not the site. Due to the nature of the interventions, participants and study staff who scheduled classes could not be masked to treatment allocation.

**Figure 1:**
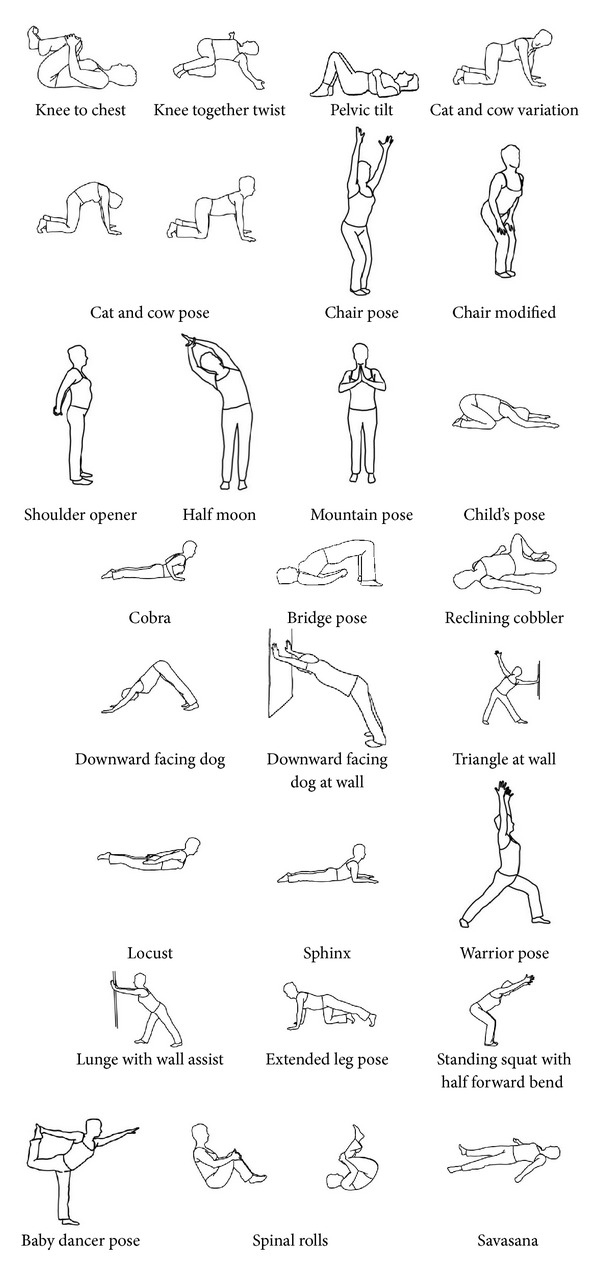
Poses used in hatha yoga protocol for chronic low back pain.

We adapted a reproducible hatha yoga protocol originally developed for our pilot study [6]. The 12-week study protocol was divided into four 3-week segments, each containing a standard set of yoga poses, breathing techniques, and relaxation exercises (see Figure 1 and Table 1). Poses increased in difficulty with each subsequent segment. The protocol also incorporated yoga philosophical principles and brief readings. Each 75-minute class followed a similar format: check-in, yoga philosophy, meditation and breathing exercise, warm-up poses, yoga poses, and closing relaxation (Table 2). The protocol used for the two groups was identical; the only difference was the number of yoga classes (1 or 2) assigned per week. To accommodate a range of physical abilities, teachers used prespecified variations of poses (e.g., chair based, using wall for support) as well as props (e.g., block, strap). Instructors encouraged all participants to practice 30 minutes daily at home on nonclass days. For home practice, participants received an audio CD of the protocol, handbook illustrating the protocol, yoga mat, strap, and block. Both groups could continue to receive routine medical care, medications, and any other ongoing cLBP treatments. Participants were discouraged, however, from starting any new nonstudy cLBP treatments, unless recommended by their physician.

Seven yoga instructors taught 17 weekly yoga classes Monday through Saturday across six different health center sites. All yoga instructors had completed at least 200 hours of yoga teacher training and had two years of teaching experience. They received a detailed teaching manual and completed 12 hours of in-person training from a senior instructor who had taught in the pilot study [6]. Class size varied (range 3–18, median 8) depending upon the class location and time. One or more instructors taught each class to ensure a participant: instructor ratio of approximately 4 : 1. Instructor availability determined their class assignments. Although we assigned participants to specific class times and locations based on their schedules and where they lived or worked, they could attend different classes when necessary. Classes included participants from both dosing groups. The principal investigator (RBS) or a senior yoga teacher observed approximately 10% of classes to assess protocol fidelity using a checklist. Participants received $25 gift cards at 3, 6, 9, and 12 weeks for participation in the study.

### 2.4. Outcomes and Follow-Up

Participants provided all data, with the exception of height and weight, through paper questionnaires administered and collected in-person by research staff at the various sites. Yoga instructors had no role in data collection other than documenting class attendance. At baseline, we collected sociodemographics, back pain history, previous cLBP treatments, past yoga use, and baseline outcome measures. Primary outcome measures were change from baseline to 12 weeks: (1) average low back pain intensity for the previous week measured on an 11-point numerical rating scale [23, 24] and (2) back-related function using the modified Roland-Morris Disability Questionnaire (RMDQ, 0–23 with higher scores reflecting poorer function) [25, 26]. Secondary outcomes included treatment adherence (defined *a priori* as attending ≥75% of recommended classes), pain medication use in the previous week (yes/no), health-related quality of life (SF-36) [27], overall improvement (7-point Likert scale, 0 = “extremely worsened,” 6 = “extremely improved”), and patient satisfaction (5-point Likert scale, 1 = “very satisfied,” 5 = “very dissatisfied”) [28].

To avoid bias from any acute effect of a yoga class, data collection occurred at least 24 hours after class. We also asked participants to complete and return weekly home practice logs, noting the number of minutes practiced outside of class each day. We elicited adverse event reports through weekly logs, prompting at classes, encouragement to notify staff of adverse events, and questionnaire items. Research staff entering data from questionnaires into StudyTrax were blinded to participants' identity and group allocation. All data were double entered and verified.

### 2.5. Statistical Analysis

We compared baseline demographics, clinical characteristics, and outcome measures using *t*- and chi-square tests. Baseline outcome measures and variables that differed between treatment groups (*P* < 0.20) were considered potential confounders. For the primary analysis of pain and function, we calculated change scores by subtracting baseline values from 12-week values. We then compared mean change scores for each treatment group using a two-sample *t*-test. We also used linear regression to adjust for any potential confounders. Assessment of a clustering effect based on study site and yoga teacher was also performed using random effects models. We similarly analyzed secondary continuous outcomes. To compare pain medication use, we used logistic regression with indicators for treatment assignment and adjustment for possible confounders, including baseline medication use. Use of specific analgesic categories (e.g., nonsteroidal anti-inflammatory drugs, acetaminophen, and opiates) was similarly compared. Treatment adherence and adverse events were compared between groups using Fisher's exact test. We used participants' home practice logs to calculate the average number of minutes of home practice per week. To estimate total yoga home practice over the entire study, we multiplied the average minutes practiced per week by 12. If a participant did not return one or more logs, we assumed the amount of home practice for those weeks was equivalent to the average calculated from returned logs. We also assessed for effect modification by categorizing home practice into tertiles and examining its interaction with group assignment via a two factor ANOVA model.

The primary analytic approach used intention to treat with any missing 12-week data replaced with 6-week values. We also performed per protocol analyses, including only participants who were adherent. We used SAS v9.2 (SAS Institute, Cary, NC) and an *α* = 0.05 criterion for significance. Our planned sample size of 96 had adequate power (80%) to detect minimal clinically important differences between groups in pain (1.5-points) [29, 30] and RMDQ (3.0-points) [3, 31] assuming a two-sided *α* = 0.05, standard deviations of 2.3 for pain and 4.7 for RMDQ [3], and a liberal 20% drop-out rate.

To explore the robustness of our primary outcome findings, we also assessed posthoc the proportion of participants in each group achieving ≥30% and ≥50% improvement from baseline, often considered to correspond to minimal and substantial clinical significant change, respectively [30]. Using data from all participants, we also explored posthoc the relationship between change in primary outcomes and total yoga classes attended using a nonparametric locally weighted scatterplot smoother (LOESS) [32].

## 3. Results

### 3.1. Study Participants

**Figure 2:**
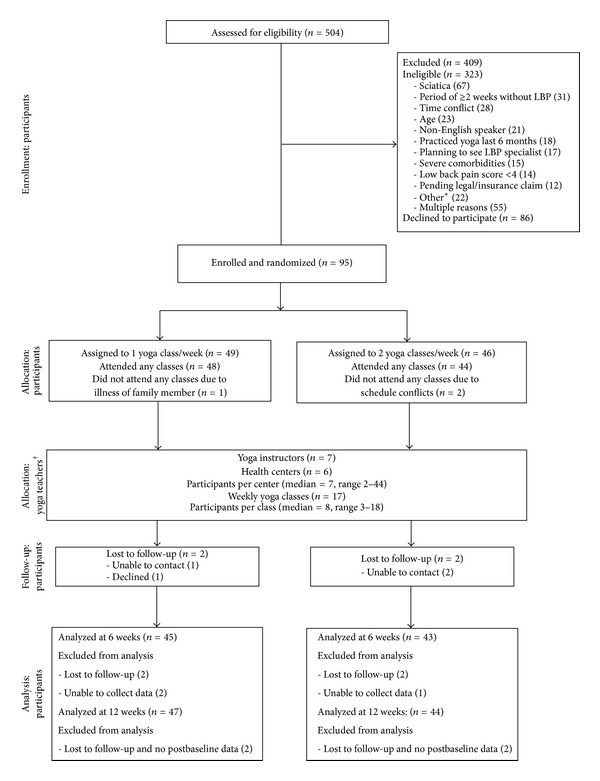
Participant flow diagram. *Other reasons for ineligibility: alcohol abuse (6), low back surgery within 3 years (4), specific LBP pathology (4), pregnancy (3), unexplained weight loss (2), drug abuse (1), being wheelchair dependent (1), and unwilling to travel (1). ^†^Participants randomized to either once-weekly or twice-weekly groups could participate in any of the 17 yoga classes.

Figure 2 is our study flow diagram.  Table 3 shows baseline characteristics of the 95 participants. Less than 20% were white and one-third had a high school education or less. Over three-fourths had annual household incomes less than $40,000. Mean baseline back pain intensity (7.1 and 6.7 for once- and twice-weekly, resp.) and function (13.7 versus 13.6) were consistent with moderate to severe low back pain. Past use of conventional and CAM cLBP treatments was common. Few participants practiced yoga previously. The two groups were similar on all other outcome variables and most baseline characteristics.

### 3.2. Interventions

Participants assigned to once-weekly and twice-weekly yoga classes attended a median of 10 and 16 classes, respectively. Thirty-two (65%) and 20 (44%) participants assigned to once-weekly and twice-weekly classes, respectively, achieved treatment adherence (*P* = 0.040). Eighty-five of 95 participants returned at least one home practice log, with a median of nine home practice logs among all participants. Seven participants returned all 12 weekly home practice logs. Participants in both groups practiced at home for a median of four days per week. Estimated weekly amount of home practice was similar (93 versus 97 minutes for once- and twice-weekly groups, resp., *P* = 0.80). Twice-weekly participants performed more yoga over the entire study (classes + home practice) than members of the once-weekly group (37.0 (95% CI 30.6 to 43.5) versus 29.0 (95% CI 25.0 to 33.0) hours, resp., *P* = 0.037). We observed classes to assess treatment fidelity and found on average that 89% of yoga protocol elements (e.g., breathing exercises, poses) were correctly delivered as detailed in the protocol.

Use of other cLBP treatments during the study was reported by 26 (53%) and 28 (61%) of once- and twice-weekly participants, respectively: massage 23 (47%) versus 22 (48%), physical therapy 13 (27%) versus 10 (22%), acupuncture 8 (16%) versus 10 (22%), chiropractic 7 (14%) versus 6 (13%), and epidural injections 4 (8%) versus 4 (9%).

### 3.3. Primary Outcomes

**Figure 3:**
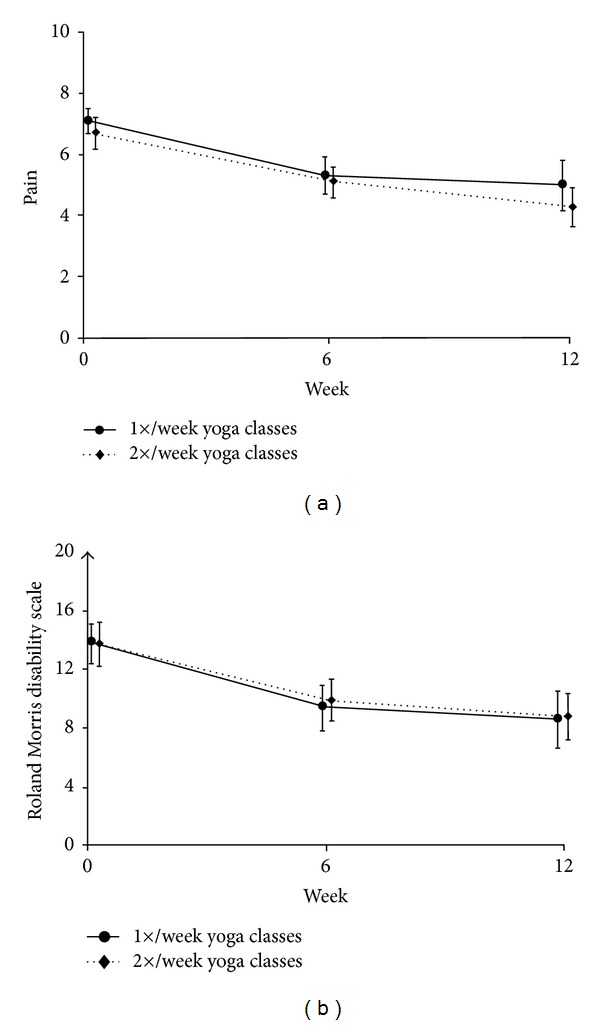
Mean pain and RMDQ scores over time, by randomly assigned group. Results are unadjusted; adjustment for potential confounders (race, education, cLBP duration, satisfaction with previous back care, history of yoga use, and baseline outcome measurements) resulted in essentially similar findings. Bars indicate 95% confidence intervals. RMDQ: modified Roland Morris Disability Questionnaire (0–23 with higher scores reflecting worse back pain-related function). (a) Mean low back pain intensity in the previous week on an 11-point numerical rating scale. (b) Mean RMDQ scores.

Figure 3 shows unadjusted mean pain and RMDQ scores at baseline, 6 and 12 weeks. Both once- and twice-weekly classes showed clinically meaningful and statistically significant (*P* < 0.001) decreases from baseline in pain at 12 weeks (Table 4): −2.1 (95% CI −2.9 to −1.3) and −2.4 (95% CI −3.1 to −1.8), respectively. Back-related function also improved for the once- and twice-weekly groups at 12 weeks: −5.1 (95% CI −7.0 to −3.2) and −4.9 (95% CI −6.5 to −3.3), respectively. However, there were no statistically significant differences between the two groups in pain or function. Adjustment for potential confounders (race, education, cLBP duration, satisfaction with previous back care, previous yoga use, and baseline outcome measurements) did not result in any meaningful changes in our results (details available from authors). We also found no evidence of clustering by study site or yoga instructor, making an adjustment for nonindependent outcomes unnecessary.

### 3.4. Other Outcomes

**Figure 4:**
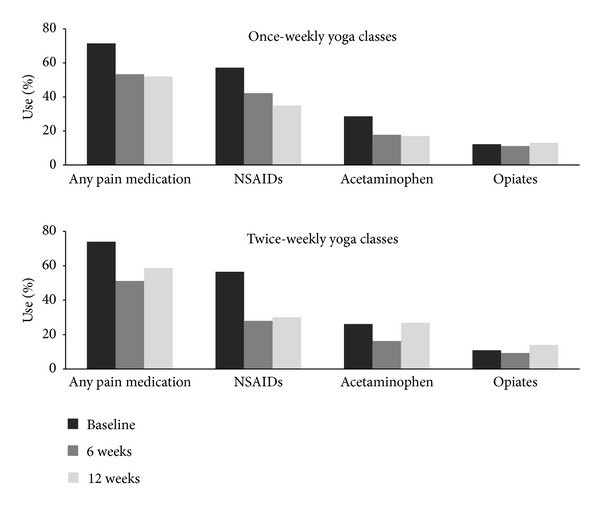
Pain medication use over time, by randomly assigned group. Height of bars indicates percentage of participants reporting any use within the previous week. NSAIDs: nonsteroidal anti-inflammatory drugs.

Table 4 also demonstrates that once-weekly and twice-weekly groups had within-group statistically significant improvements (*P* < 0.001) at 12 weeks in SF-36 physical health (6.4 and 6.3, resp.). SF-36 mental health improved only within the once-weekly group. However, overall SF-36 changes were modest and did not differ between groups. Use of any pain medication at 6 weeks decreased by 27% and 35% in the once- and twice-weekly groups, respectively, and remained similar at 12 weeks (Figure 4). Use of NSAIDs decreased in both groups. Opiate use did not significantly change. There were no statistically significant between-group differences in use of any pain medication or specific analgesic categories. The two groups reported the same overall improvement scores (mean 4.5, median 5) and nearly identical satisfaction scores (mean 1.3 versus 1.5 for once- versus twice-weekly, resp.; median 1 for both).

Per protocol analysis of change in pain from baseline to 12 weeks also did not show any statistical significant difference between the once-weekly (−2.4 (95% CI −3.4 to −1.4)) and twice-weekly (−2.7 (95% CI −3.7 to −1.7)) groups (*P* = 0.71). Although the back-related function per protocol analysis revealed greater change for the twice per week group (−7.7 (95% CI −9.9 to −5.6) versus −5.3 (95% CI −7.6 to −3.0)), this did not reach statistical significance (*P* = 0.14). Per protocol analyses of our secondary outcomes also did not demonstrate any statistically significant differences between groups.

Similar proportions of participants in the once- and twice-weekly groups experienced ≥30% improvement from baseline for the primary outcomes (23 (49%) versus 26 (59%) for pain (*P* = 0.33); 27 (57%) versus 29 (66%) for RMDQ (*P* = 0.41)). This was also the case for participants with ≥50% improvement (17 (36%) versus 14 (32%) for pain (*P* = 0.66); 22 (47%) versus 22 (50%) for RMDQ (*P* = 0.76)).

**Figure 5:**
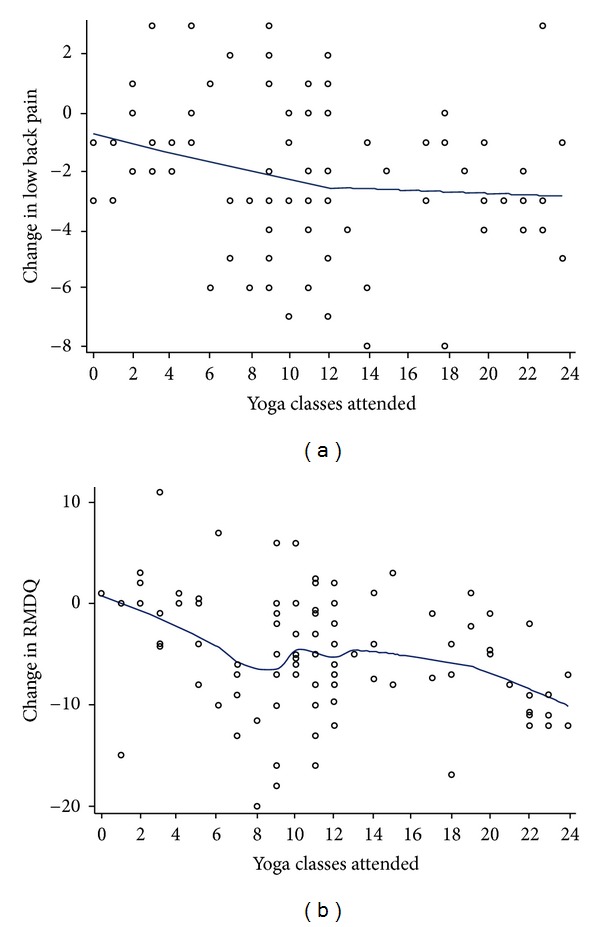
Dose-response relationship between yoga classes attended and change in primary outcomes, entire sample. (a) Yoga class attendance and change in mean low back pain intensity from baseline to week 12. (b) Yoga class attendance and change in mean RMDQ score from baseline to week 12.

Figure 5 explores the relationship for all study participants between total yoga classes attended and change in back pain intensity and function. Although there is substantial variation in the data, a modest dose-response relationship may be present. The slope of the relationship appears to decrease after approximately 12 classes for pain and 9 classes for the RMDQ.

### 3.5. Adverse Events

Thirty adverse events occurred in 13 (27%) and 15 (34%) participants in the once- and twice-weekly groups, respectively (*P* = 0.47).  Table 5 shows that most adverse events were musculoskeletal pain episodes, with low back pain exacerbation being most common. The majority were self-limited and deemed by the investigators and data safety monitoring board to not be serious or definitely related to the yoga intervention. In either group, individuals reporting adverse events did not attend fewer classes or do less home practice than participants without adverse events (see footnote, Table 5).

## 4. Discussion

In our 12-week randomized dosing trial comparing once- and twice-weekly yoga classes, 95 predominantly minority low income adults with moderate to severe chronic low back pain experienced clinically significant but statistically similar improvements in pain and back-related function. Adverse events were common in both groups; however most were self-limited, not definitely related to yoga, and not serious. Our findings suggest yoga's effectiveness for cLBP is generalizable to racially diverse, low income, and more severely impaired patients. Moreover, twice-weekly classes do not appear to offer additional benefit and are more difficult to comply with.

Several factors may explain why twice-weekly classes were not more effective than once-weekly classes. First, participants in the twice-weekly group were less likely than once-weekly participants to be adherent. Secondly, home practice in both groups was similar. If home practice contributes to improvement, then the total effective dose of yoga (estimated time spent in classes *plus* home practice) differed only by 28% (37 versus 29 hours for once- and twice-weekly, resp.). Lastly, both groups experienced most of their benefit by six weeks (Figure 3), suggesting that an effective dose for yoga in cLBP may be as little as six weekly classes augmented by home practice. A much greater twice-weekly dose of 24 classes over 12 weeks may not therefore provide substantial marginal benefit over the once-weekly dose. Whether a six-week program is sufficient for long-term maintenance and effectiveness is unknown and requires further study.

The improvement in pain and function observed for both yoga doses are consistent with findings from the largest yoga for chronic low back pain trials. Our participants improved by approximately 2 and 5-points for pain and function, respectively, compared to 1.6 and 5.2-points in Sherman's YES trial [3]. Yoga participants in Tilbrook's UK study improved 2.2-points in function [4]. However, our sample's baseline back pain intensity (6.9) and function scores (13.9) were more severe than in the YES trial (4.7 and 9.1, resp.) and in Tilbrook's study (RMDQ 7.8, pain score not reported) [4]. Our participants' higher baseline rates of pain medication use (73%) and below average physical and mental SF-36 scores also reflect their substantial morbidity at study entry.

The disparity in baseline pain and impairment between our sample and other trials is likely related to different sociodemographic characteristics. Sherman's trial recruited participants from a Pacific Northwest integrated healthcare organization who were predominantly white, employed and had incomes greater than $45,000 [3]. Tilbrook's UK sample was also mostly employed; race and income were not reported [4]. By contrast, our participants were mostly non-white, unemployed or disabled, and had incomes less than $40,000.

Although back pain prevalence in US whites and blacks is similar [33], racial disparities in access, treatment, and pain perception exist. For example, medical expenditures for low back pain in minorities are 30% lower than for whites [34]. Minorities with low back pain also receive less patient education [35], specialty referrals [36], pain medication [37], and intensive rehabilitation for occupational back injuries [38]. A history of racial discrimination by a minority individual can also be associated with greater levels of back pain [39].

Population-based studies of yoga use in the USA have shown yoga users are usually white in higher socioeconomic groups [40, 41]. In 2002, blacks were 34% less likely to use yoga compared to whites. Adults lacking any college education were 62% less likely to be yoga users. However, our study suggests that low income minority populations will accept and be satisfied with a yoga program, at least in the context of a clinical trial that offers financial compensation and free yoga classes. The cost of individual community yoga classes, typically ranging $15–$20, may be prohibitive for these populations. Structured yoga programs for chronic low back pain need to be implemented in community and healthcare settings and evaluated to ascertain their feasibility and acceptance.

Limitations of our study include those common to nonpharmacologic trials for cLBP, including inability to blind participants to their treatment assignment and the use of self-report measures. Other limitations include the lack of a non-yoga control group, differential adherence, high use of nonstudy treatments, and no long-term follow-up. The rationale for only measuring short-term outcomes related to the study's purpose, that is, to efficiently inform the yoga class frequency for a subsequent larger and longer trial where long-term follow-up data will be collected. Accurate assessment of home practice and its contribution to dose is limited due to potential self-report bias and incomplete reporting. More reliable methods are needed to measure home practice and determine its importance. In addition, due to decreased sample size, the per protocol analyses were underpowered to detect statistically significant between group differences for pain (54% power) and back-related function (73% power). However, the observed differences between groups were still smaller than the stated minimal clinically important differences. Whether a larger study with more patients adherent to the two dosing protocols would show superiority for the twice-weekly group is unknown. Regarding the posthoc dose-response analyses, the data were drawn from the entire sample and had large variability, and therefore any conclusions about the causality of the association between number of classes and response is not possible.

## 5. Conclusion

In a predominantly minority underserved population with moderate to severe chronic low back pain, 12 weeks of once-weekly yoga classes were similarly effective as twice-weekly classes. In conjunction with the convenience and lower expense of once-weekly classes, these data provide clinicians practical information about the minimum number of classes per week they should recommend to patients interested in trying yoga for their chronic low back pain. 

## Figures and Tables

**Table 1:** Twelve-week standardized hatha yoga protocol for the treatment of chronic low back pain.

Yoga Posture	Protocol segments				Total weeks Incorporating posture
	Segment 1 Weeks 1–3	Segment 2 Weeks 4–6	Segment 3 Weeks 7–9	Segment 4 Weeks 10–12	
	Opening to Something Greater	Listening to the Wisdom of the Body	Engaging Your Power	Bringing it Home	
*Savasana* relaxation and breathing exercises	*✓*	*✓*	*✓*	*✓*	12
Warm-up postures					
Knee to chest*	*✓*	*✓*	*✓*	*✓*	12
Knee together twist*	*✓*	*✓*	*✓*	*✓*	12
Pelvic tilt*	*✓*	*✓*	*✓*	*✓*	12
Cat and cow (and modifications)*	*✓*	*✓*	*✓*	*✓*	12
Chair (and modified)*	*✓*	*✓*	*✓*	*✓*	12
Shoulder opener*	*✓*	*✓*	*✓*		9
Half moon*	*✓*	*✓*	*✓*	*✓*	12
Mountain (and modifications)*		*✓*	*✓*	*✓*	9
Yoga postures					
Child's pose*	*✓*		*✓*	*✓*	9
Cobra (and modified)*	*✓*	*✓*	*✓*	*✓*	12
Bridge*	*✓*	*✓*	*✓*		9
Reclining cobbler	*✓*	*✓*	*✓*		9
Downward facing dog (and at wall)*		*✓*		*✓*	6
Triangle pose at wall		*✓*			3
Locust pose		*✓*			3
Sphinx*		*✓*	*✓*	*✓*	9
Warrior*			*✓*	*✓*	6
Lunge with wall assist			*✓*	*✓*	6
Extended leg*			*✓*	*✓*	6
Standing squat with half forward bend				*✓*	3
Baby dancer				*✓*	3
Spinal rolls				*✓*	3
*Savasana* ending relaxation	*✓*	*✓*	*✓*	*✓*	12

The same 12-week hatha yoga protocol for chronic low back pain was used in both groups. The only difference was in the number of 75-minute classes per week offered to participants (once-weekly versus twice-weekly). The protocol provided variations of poses to accommodate different abilities.

*Exercises included on the audio CD provided to participants for home practice.

**Table 2:** Standard yoga class format.

Curriculum elements	Time (min)
Check in with participants	5
Centering, yoga philosophy*, and lesson introduction	10
Relaxation	5
Breathing exercise	5
Warm ups	15
Yoga postures	25
Integrative relaxation	5
Closing	5

Total time	75 minutes

*A standardized set of yoga philosophical principles were introduced, such as nonviolence, gratitude, moderation, and self-acceptance.

**Table 3:** Baseline characteristics of 95 adults with chronic low back pain randomized to once- or twice-weekly hatha yoga classes*.

	1 class/week (*n* = 49)	2 classes/week (*n* = 46)	Total (*n* = 95)
Mean age, years (SD)	46.4 (11.1)	48.7 (10.3)	47.5 (10.7)
Female	35 (71)	37 (80)	72 (76)
Race^||^			
White	5 (10)	12 (26)	17 (18)
Black	33 (67)	19 (41)	52 (55)
Other	11 (22)	15 (33)	26 (27)
Hispanic	3 (6)	6 (13)	9 (10)
US born^†^	39 (80)	36 (78)	75 (79)
Language spoken at home			
English	43 (88)	38 (83)	81 (85)
Spanish	2 (4)	4 (9)	6 (6)
Haitian Creole	3 (6)	1 (2)	4 (4)
Other^‡^	1 (2)	3 (7)	4 (4)
Education^||^			
High school or less	21 (43)	12 (26)	33 (35)
Beyond high school	28 (57)	34 (74)	62 (65)
Employment			
Employed	21 (43)	21 (46)	42 (44)
Unemployed	17 (35)	13 (28)	30 (32)
Disabled	8 (16)	10 (22)	18 (19)
Other/missing	3 (6)	2 (4)	5 (5)
Income			
≤$10,000	11 (22.5)	13 (28)	24 (25)
$10,001–$20,000	11 (22.5)	11 (24)	22 (23)
$20,001–$40,000	14 (29)	10 (22)	24 (25)
$40,001–$70,000	5 (10)	6 (13)	11 (12)
>$70,000	7 (14)	2 (4)	9 (10)
Missing	1 (2)	4 (9)	5 (5)
Health insurance			
Public	27 (55)	26 (57)	53 (56)
Private	21 (43)	20 (43)	41 (43)
None	1 (2)	0	1 (1)
Duration of LBP^||^			
<1 year	6 (12)	16 (35)	22 (23)
1–3 years	16 (33)	13 (28)	29 (31)
4–9 years	13 (27)	10 (22)	23 (24)
≥10 years	13 (27)	7 (15)	20 (21)
Missing	1 (2)	0	1 (1)
Sciatica	18 (37)	15 (33)	33 (35)
Previous LBP treatments			
Heat/ice	37 (76)	37 (80)	74 (78)
Exercise	37 (76)	32 (70)	69 (73)
Massage	35 (71)	33 (72)	68 (72)
Physical therapy	33 (67)	28 (61)	61 (64)
Chiropractic	18 (37)	20 (43)	38 (40)
Acupuncture	13 (27)	15 (33)	28 (29)
Epidural steroid injections	9 (18)	9 (20)	18 (19)
Trigger point injections	7 (14)	6 (13)	13 (14)
Back surgery	2 (4)	4 (9)	6 (6)
Osteopathic manipulation	2 (4)	4 (9)	6 (6)
Other^§^	2 (4)	5 (11)	7 (7)
Hours exercise/week, mean (SD)	5.1 (10.0)	4.6 (5.9)	4.8 (8.2)
Previous yoga use^||^	9 (18)	3 (7)	12 (13)
BMI, mean (SD)	29.6 (7.1)	30.5 (6.3)	30.0 (6.7)
Pain intensity in previous week, mean (SD)	7.1 (1.4)	6.7 (1.8)	6.9 (1.6)
RMDQ, mean (SD)	13.7 (4.8)	13.6 (5.2)	13.7 (5.0)
Pain medication use in last week			
Any category	35 (71)	34 (74)	69 (73)
NSAIDs	28 (57)	26 (57)	54 (57)
Acetaminophen	14 (29)	12 (26)	26 (27)
Opiates	6 (12)	5 (11)	11 (12)
Other	4 (8)	7 (15)	11 (12)
SF-36 Physical Health, mean (SD)	37.5 (7.4)	37.4 (7.9)	37.4 (7.6)
SF-36 Mental Health, mean (SD)	44.8 (12.4)	44.1 (13.1)	44.5 (12.7)
Satisfaction with previous back care^||^			
Very satisfied	4 (8)	0	4 (4)
Somewhat satisfied	8 (16)	5 (11)	13 (14)
Neither satisfied or dissatisfied	20 (41)	14 (30)	34 (36)
Somewhat dissatisfied	9 (18)	10 (22)	19 (20)
Very dissatisfied	8 (16)	12 (26)	20 (21)
Missing	0	5 (11)	5 (5)
Hours/day of LBP, mean (SD)	10.1 (7.6)	9.4 (7.0)	10 (7)
Days of restricted activity due to LBP in last 4 weeks, mean (SD)	11.7 (8.9)	11.4 (9.1)	12 (9)

*Unless otherwise noted, values are the numbers (percentages) of participants.

^||^Treated as a potential confounder due to between group differences (*P* < 0.20) at baseline.

^†^Non-US born participants were from the Caribbean (Haiti (3), Dominican Republic (2), Barbados (2), Jamaica (2), Aruba, St. Kitts, St. Vincent), Africa (Cape Verde (2), Nigeria, Uganda, Liberia), India (2) and Missing (1).

^‡^Other languages spoken at home included Portuguese Creole, Edo, Bengali, and Gujarati.

^§^Other therapies used in the past for back pain: meditation (2), hot tub (2), relaxation (1), deep breathing (1), tai chi (1), sauna (1), stretching (1), and corset (1).

Abbreviations: BMI: body mass index; SD: standard deviation; LBP: low back pain; NSAIDs: nonsteroidal anti-inflammatory drugs; RMDQ: modified Roland Morris Disability Questionnaire; SF-36: the Short Form-36 Health Survey.

**Table 4:** Outcome measurements at 6 and 12 weeks.

Outcome measure	Mean baseline value (SD)	Mean change from baseline (95% CI)			
		6 weeks	*P* value	12 weeks	*P* value
Pain					
Once-weekly classes	7.1 (1.4)	−1.8 (−2.5 to −1.2)*		−2.1 (−2.9 to −1.3)*	
Twice-weekly classes	6.7 (1.8)	−1.5 (−2.1 to −1.0)*		−2.4 (−3.1 to −1.8)*	
Between-group difference in means		−0.3 (−1.1 to 0.6)	0.49	0.3 (−0.2 to 0.8)	0.62
RMDQ					
Once-weekly classes	13.7 (4.8)	−4.4 (−6.0 to −2.8)*		−5.1 (−7.0 to −3.2)*	
Twice-weekly classes	13.6 (5.2)	−3.8 (−5.2 to −2.4)*		−4.9 (−6.5 to −3.3)*	
Between-group difference in means		−0.6 (−2.7 to 1.6)	0.62	−0.1 (−1.4 to 1.2)	0.83
SF-36 Physical					
Once-weekly classes	37.5 (7.4)	6.7 (4.1 to 9.4)*		6.4 (3.6 to 9.2)*	
Twice-weekly classes	37.4 (7.9)	5.1 (3.2 to 7.0)*		6.3 (4.1 to 8.4)*	
Between-group difference in means		1.6 (−1.6 to 4.9)	0.33	0.2 (−3.4 to 3.7)	0.93
SF-36 Mental					
Once-weekly classes	44.8 (12.4)	4.8 (1.9 to 7.7)^†^		4.0 (1.3 to 6.7)^‡^	
Twice-weekly classes	44.1 (13.1)	2.6 (−0.4 to 5.6)		2.5 (−0.7 to 5.7)	
Between-group difference in means		2.2 (−1.9 to 6.3)	0.29	1.5 (−2.6 to 5.6)	0.47

All analyses are unadjusted and performed using the intent to treat principle. After controlling for potential confounders (race, education, duration of chronic low back pain, satisfaction with previous back care, and previous yoga use) and baseline outcome measurements, adjusted results were nearly identical to the unadjusted results.

Abbreviations: SD: standard deviation; RMDQ: modified Roland Morris Disability Questionnaire; SF-36: the Short Form-36 Health Survey.

*P < 0.0001 for within-group difference compared to baseline.

^†^ *P* = 0.002 for within-group difference compared to baseline.

^‡^ *P* = 0.005 for within-group difference compared to baseline.

**Table 5:** Adverse events*.

Adverse Event, *n*	1 class/week (*n* = 49)	2 classes/week (*n* = 46)
Back pain	5	8
Neck pain	1	3^†^
Sciatica	1	2
Headache	1	2
Dizziness	1	1
Knee pain	1	0
Ankle pain	0	1
Shoulder pain	1	0
Abdominal pain	1	0
Wheezing	1	0
*Total adverse event reports *	13	17
Related to intervention		
Definitely	1	2
Possibly	12	15
Serious^‡^	0	1

*Average number of classes attended in the once-weekly group was 8.6 versus 8.2 (*P* = 0.73) for those with and without adverse events, respectively. The average number of classes attended in the twice-weekly group was 15.5 versus 12.7 (*P* = 0.21) for those with and without adverse events, respectively. Mean home practice in the once-weekly group was 942 versus 1192 minutes (*P* = 0.27) for those with and without adverse events, respectively. Mean home practice in the twice-weekly group was 1224 versus 1104 minutes (*P* = 0.68) for those with and without adverse events, respectively.

^†^Included one participant judged to have a serious adverse event due to persistent symptoms of cervical radiculopathy, possibly from hyperextension of the neck during cow pose in the setting of preexisting cervical disc disease.

^‡^Serious adverse events were defined as any adverse event that resulted in one or more of the following outcomes: death, life-threatening event, inpatient hospitalization, and persistent or significant disability/incapacity; congenital anomaly; or an important medical event based upon appropriate medical judgment.
